# In silico investigation on interaction of small Ag_6_ nano-particle cluster with tyramine neurotransmitter

**DOI:** 10.1038/s41598-023-45847-0

**Published:** 2023-11-18

**Authors:** Subhendu Chakroborty, E. Shakerzadeh, T. Yadav, Nilima Priyadarsini Mishra, Arundhati Barik, Versha Upadhyay, Abhilasha, Siba Soren, Jitendra Malviya, Amiya Ranjan Panda, Kartik Uniyal, Narendra Kumar, Shradha Wagadre, F. P. Pandey

**Affiliations:** 1 Department of Basic Sciences, IITM, IES University, Bhopal, Madhya Pradesh 462044 India; 2 Chemistry Department, Faculty of Science, Shahid Chamran University of Ahvaz, Ahvaz, Iran; 3 Department of Chemistry, Indian Institute of Technology Ropar, Nangal Road, Rupnagar, 140001 Punjab India; 4 CIPET: Institute of Petrochemicals Technology [IPT], Bhubaneswar, Odisha India; 5 Botany Department, Maya Group of Colleges Dehradun, Dehradun, India; 6 Dolphin PG Institute of Biomedical and Natural Sciences, Manduwala, Dehradun, Uttrakhand India; 7 Department of Chemistry, Govt. Women’s College, Baripada, 757001 India; 8 Department of Life Sciences & Biological Sciences, IES University, Bhopal, India; 9 Kabi Samrat Upendra Bhanja (KSUB) College, Bhanjanagar, Ganjam, Odisha India; 10 Department of Biochemistry and Biotechnology, Sardar Bhagwan Singh University Balawala, Dehradun, 248161 Uttrakhand India; 11 Alpine Institute of Management and Technology Dehradun (Uttarakhand), Dehradun, India; 12 Scitechesy Research and Technology Private Limited, Central Discovery Center, Banaras Hindu University, Varanasi, Uttar Pradesh India

**Keywords:** Computational biology and bioinformatics, Chemistry, Nanoscience and technology

## Abstract

The interaction of tyramine neurotransmitter with silver nano-particle (Ag_6_) cluster is explored in terms of the molecular structure, electronic properties and NBO analysis of tyramine-AgNPs bio-molecular conjugate. The adsorption mechanism of tyramine onto the Ag_6_ cluster has been investigated through computing of the electronic and geometrical properties in addition to the adsorption energies in various possible configurations. The magnitude of adsorption energy corresponding to the most favorable tyramine-Ag_6_ bio-molecular conjugate has been computed to be − 14.36 kcal/mol in the gas phase, which infers a good adsorption of tyramine with AgNPs cluster suggesting the practical applications of tyramine-AgNPs bio-molecular conjugates in bio-sensing, drug delivery, bio-imaging and other applications. Different electronic properties such as the energy gap of HOMO–LUMO, Fermi level and work function have been investigated in detail. Moreover, the effect of aqueous media on adsorption energy and electronic properties of the most favorable tyramine-AgNPs bio-molecular conjugate is investigated in order to understand the impact of the real biological situation.

## Introduction

The use of nanoparticles (NPs) in biological and industrial applications such as bio-imaging, medication administration, plasmonics, photonics, chemical sensors, anti-microbial activities, cell electrodes, optical devices, anti-microbial coating and hyperthermia therapy is being investigated progressively^1–5^. Thus, the experimental and theoretical studies concerning the interaction of nanoparticles with biological systems including nucleic acids, neurotransmitters, biomolecules, peptides, and proteins are very crucial in order to have a better understanding of the adsorption mechanism of aforementioned biological systems over the NPs surface. This can help in safe and proficient biological and industrial application of nanoparticles based bio-molecular conjugates. Among various NPs, the silver nano-particles (AgNPs) have drawn great attention from the scientific community and gaining popularity as suitable nanomaterials for biological applications owing to their remarkable properties including good biocompatibility with biological molecules (i.e. amino acid, peptides and nucleic acids), non-toxicity as well as shape-dependent electronic and optical features^6–10^. Furthermore, the AgNPs have unique optical, electrical, and thermal properties and a high surface-to-volume ratio that make them ideal for cutting-edge applications in the fields of medicine, biology, mechanics, packaging, food science, electronics, and information technology, among others^11^.

Tyramine is an essential trace biogenic amine and acts as a neurotransmitter^12^. Trace biogenic amines play an important role in many human disorders that involve mood, emotion, cognition, and attention. Tyramine is being synthesized by the mammalian brain and peripheral nerve tissues^13,14^. It is found in excessive extent in assorted variety of raw and fermented foods together with fish, meat, fruits, cheese, soybean products, and wine^15^. A large amount of consumption of tyramine rich food shows toxic effect on human health by producing the symptoms like flush, rash, hypertonia, vomiting, palpitation and tachycardia. Besides, it also leads to dangerous blood pressure increase and chance of strong migraine headaches^16^. The remarkable set of properties of AgNPs and their application in biology, medicine, chemical sensors and bio-imaging and the scientific usefulness of tyramine motivated us to perform a detailed in silico investigation on the modeling of AgNPs@Tyramine bio-molecular conjugate to have a deep insight into the molecular interaction of neurotransmitter with AgNPs cluster and its electronic properties. The interaction of AgNPs cluster and tyramine can be a crucial perquisite in drug delivery applications of tyramine related diseases and many other future industrial and biological investigations.

Literature survey revealed that the interaction of silver nano-particles (AgNPs) with biological systems have been studied by a number of authors in many different aspects^6,17–24^. Ando and coworkers used silver, gold, and silver-gold alloy as imaging probe to trail motions of isolated biomolecules^17^. In order to track the motion of isolated biomolecules, they designed the multicolor high-speed single particle tracking system of silver, gold, and silver-gold alloy. The interaction of silver nano-particles with monosaccharide was performed with the help of density functional theory by Gallegos and coworkers in order to explore a better reducing agent among α-Dglucose, α-d-ribose, D-erythrose, and glyceraldehyde in green synthesis approach^18^. Besides, they also performed theoretical computations on electron localization function analysis which helped in the explaining the reduction procedure in the formation of AgNPs. A comparative investigation of the interaction mechanisms between various sized, shaped, and surface functionalized silver and gold nanomaterials to commercially available human transferrin, a glycosylated protein, and to its nonglycosylated recombinant form was reported by Barber et al.^19^. Fahy and his co-workers used silver-engineered nanomaterials in evaluation of surface charge role in protein interactions and cellular cytotoxicity^20^. The report on kinetic study of AgNPs under acidic conditions and impact of protein interactions i.e., bovine serum albumin (BSA) to the colloidal stability of silver nano-particles was made by Tai et al.^21^. A detailed study on the interaction of ionic liquids with AgNPs was reported by Banjare and his group^22^. It was reported that AgNPs could be effective drug carriers for anti-malarial drugs for treatment of malaria and Covid-19 by performing theoretical calculations at DFTB3LYP/6-311++ g(d,p) level^6^. Venkatesh and coworkers reported the geometrical, vibrational and physicochemical properties of 2-deoxy-D-glucose by performing the DFT calculations on its interaction with AgNPs^23^. Moreover, Jadoon with the help of theoretical calculations showed that silver (Ag_6_) decorated Coronene quantum dot is an effective non-enzymatic sensor for glucose and H_2_O_2_ detection and in another study they proposed silver cluster decorated graphene nanoflakes can be an effective detector for detection of nitroaniline isomers^24,25^. Adsorption of p-aminophenol over silver-graphene composite was explored by Ayub and co-workers^26^. DFT study on the sensitivity of silver-graphene quantum dots for vital and harmful analytes were also performed^27^. Moreover, silver cluster based sensors has been proposed to expose various other molecules as well^28,29^. We have reported possible low lying energy conformers, vibrational dynamics and NBO analysis of isolated tyramine and its interaction with HCl in our earlier reported investigation^12^. Now we are extending our work on tyramine to investigate its adsorption and charge transfer mechanism over the surface of Ag_6_ nanoparticle cluster.

## Computational details

All the theoretical calculations have been performed at Gaussian09 suite^30^ by employing density functional theory (DFT) through B3LYP functional. The Lanl2DZ and 6-31G(d,p) basis sets are applied for Ag atoms and tyramine-molecule. The adsorption energies of the most favorable Ag_6_@tyramine complex have been computed as follows:

1 $$\user2{ E}_{{{\varvec{ads}}}} = {\varvec{E}}\left( {{\mathbf{Ag}}_{{\mathbf{6}}} - {\mathbf{Tyramine}}} \right) \, {-}{\varvec{E}}\left( {{\mathbf{Ag}}_{{\mathbf{6}}} } \right) \, {-}{\varvec{E}}\left( {{\mathbf{Tyramine}}} \right) \, + \left( {{\mathbf{BSSE}}} \right)$$

where, *E*(Ag_6_-Tyramine) is the energy of Ag_6_@tyramine complex. The *E*(Ag_6_) and *E*(Tyramine) terms denote the energies of relaxed Ag_6_ cluster and tyramine molecule, respectively. The BSSE which is basis set superposition error (BSSE) for the adsorption energy was computed by applying counterpoise method. Besides, the dispersion calculations which account interactions including short and long range contributions were also computed in case of the most favorable configuration of Ag_6_@tyramine bio-molecular conjugate through D3 version of *Grimme's* dispersion with the original D3 damping function. Accordingly, a negative adsorption energy implies to the thermodynamic stability of the obtained complex. Furthermore, natural bond orbital (NBO) analysis has been performed for the most favorable configuration of Ag_6_@tyramine in the gas phase at the same level using NBO3.1 program implemented in Gaussian09 suite. NBO method gives the information about the intra and intermolecular binding interaction, donor–acceptor interactions, delocalization of charge density and hyper-conjugative interaction energies of any molecular system. The second-order hyper-conjugative interaction energy (*E*^*(2*)^) has been computed using the following equation^31–34^:

2 $$\user2{ E}^{\left( 2 \right)} = {{\varvec{\Delta}}}{\varvec{E}}_{{{\varvec{ij}}}} = \user2{ q}_{{\varvec{i}}} \frac{{{\varvec{F}}_{{{\varvec{ij}}}}^{2} }}{{{{\varvec{\upvarepsilon}}}_{{\varvec{j}}} \user2{ } - {{\varvec{\upvarepsilon}}}_{{\varvec{i}}} }}$$

The *q*_*i*_ is the ith donor orbital occupancy, *ε*_*i*_ and *ε*_*j*_ are the diagonal elements (orbital energies), and *F*_*ij*_ is the off-diagonal NBO Fock Matrix element.

## Results and discussion

### Tyramine adsorption over Ag_6_ cluster

**Figure 1:**
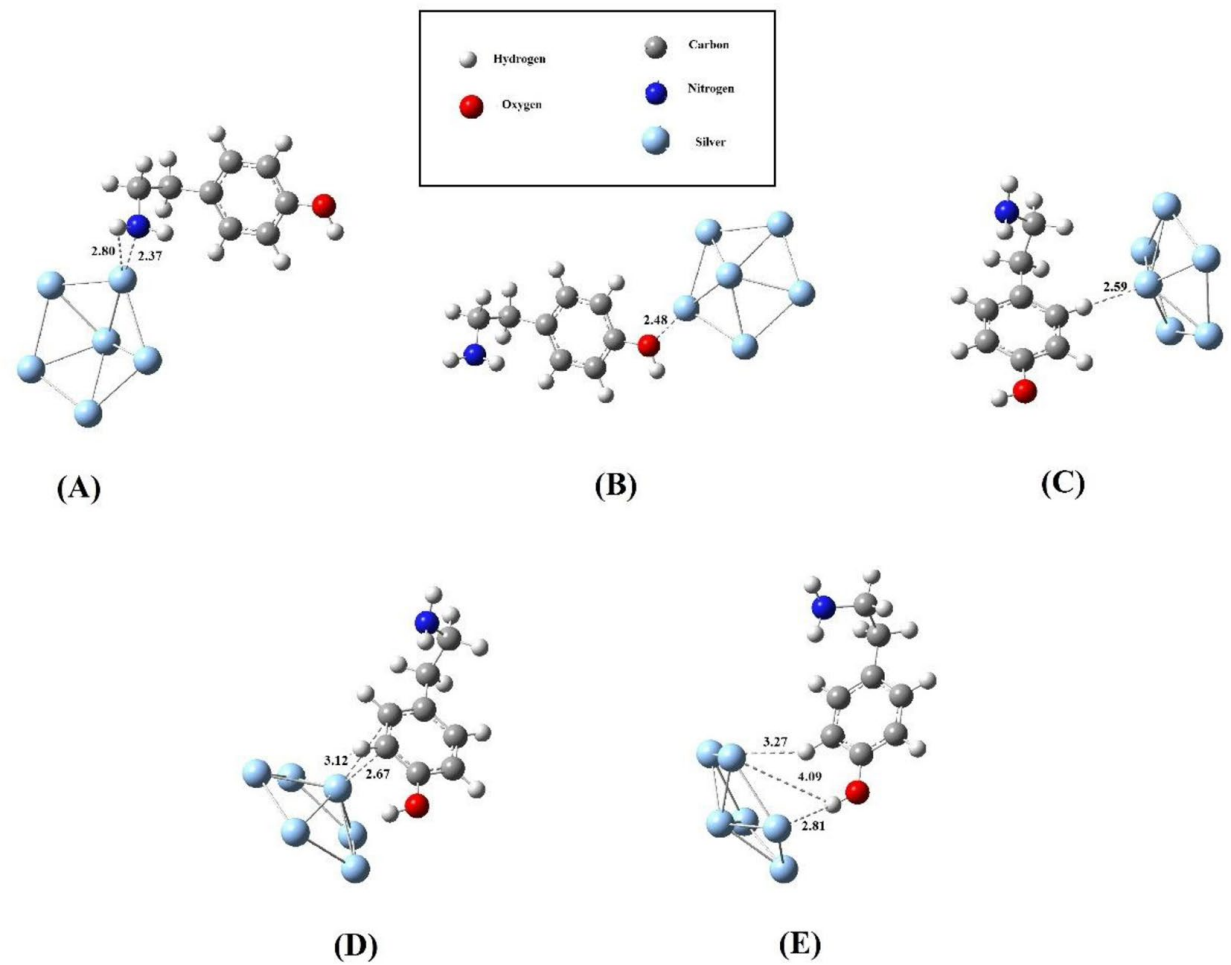
Various optimized configurations of AgNPs@tyramine.

The optimization of Ag_6_@tyramine in various possible configurations is carried out to find out the most favorable adsorption configurations of Ag_6_@tyramine bio-molecular conjugate and these optimized configurations have been used for further analysis. Consequently, five possible configurations for Ag_6_@tyramine conjugates are obtained and depicted in Fig. 1. The adsorption energy, which is very crucial for any nanomaterials based bio-molecular conjugate to utilize it practically in various biological and industrial applications is also computed for each configuration and listed in Table 1. All the five possible configurations have been simulated in gas and aqueous phase as well. Moreover, Ag_6_@tyramine bio-molecular conjugate contains even number electrons, and thereby it offers singlet multiplicity and neutral charge. Figure 1 reveals that Ag_6_ cluster interacts with amine group of side chain (i.e. in case of configuration-A) then the bond length of N-Ag and N–H…Ag are 2.37 and 2.80 Å, respectively with the adsorption energy of − 14.36 kcal/mol (without BSSE term) and the BSSE is computed to be 3.12 kcal/mol. Thus including BSSE term the adsorption energy is − 11.24 kcal/mol for this configuration. Moreover, the adsorption energy for this configuration after applying D3 version of Grimme’s dispersion with the original D3 damping function is computed to be − 21.65 kcal/mol. In configuration B, the interaction of oxygen atom (O) of hydroxyl group with Ag_6_ cluster results − 6.01 kcal/mol adsorption energy, which demonstrates that tyramine molecule, interacts more effectively through amine group with Ag_6_ cluster rather than OH group. The interaction distance between oxygen atom (O) and Ag is computed to be 2.48 Å in configuration B. In case of configurations-C, D and E the adsorption energies are computed to be − 1.02, − 5.35, and − 3.21 kcal/mol, respectively. The prominent interaction distances between different atoms of tyramine and Ag atoms have been shown in Fig. 1. A literature survey^35–40^ reveals that the magnitude of adsorption energy neither should be very high nor very low as desorption of drugs from nanomaterials can occur easily which is very necessary in drug delivery and sensing applications. The magnitude of adsorption energy in case of the most favorable configuration of Ag_6_@tyramine bio-molecular conjugate is good to utilize it in various biological and industrial applications. Moreover, the charge transfer between Ag_6_ cluster and tyramine is also computed using NBO approach for the most favorable adsorption configuration (i.e. A). NBO calculations indicate that there is a charge transfer of 0.46*e* from tyramine to Ag_6_ cluster. Thus, the direction of charge transfer is from drug to cluster and the obtained complex is stabilized through charge transfer process.

**Table 1:** Value of adsorption energies for various configurations of AgNPs@tyramine.

Configurations	E_ads_ (kcal/mol)
AgNPs@tyramine-1	− 14.36
AgNPs@tyramine-2	− 6.01
AgNPs@tyramine-3	− 1.02
AgNPs@tyramine-4	− 5.35
AgNPs@tyramine-5	− 3.21

**Figure 2:**
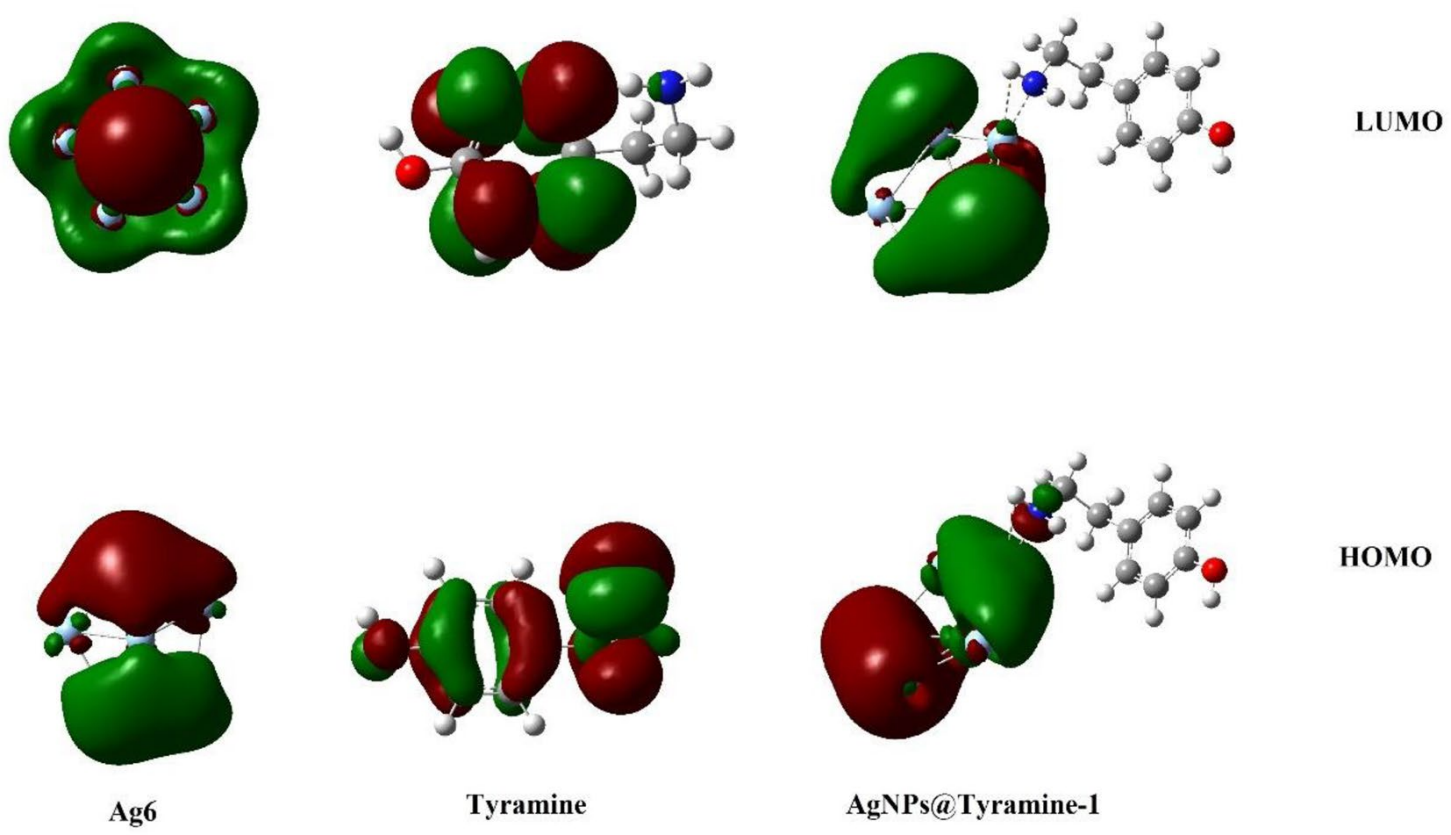
HOMO and LUMO surfaces of AgNPs cluster, Tyramine and AgNPs@tyramine.

Some important electronic parameters viz HOMO energy (E_H_)_,_ LUMO energy (E_L_), energy gap (E_g_) and Fermi level (E_F_) for Ag_6_ cluster before and after tyramine adsorption are summarized in Table 2. The study of highest occupied molecular orbital (HOMO) and lowest unoccupied molecular orbital (LUMO) are very crucial in quantum chemistry as these provides details about electronic characteristics of molecular species^41^. Furthermore, graphical representation of HOMO and LUMO iso-surfaces is depicted in Fig. 2.

**Table 2:** Calculated HOMO energy (E_H_), LUMO energy (E_L_), HOMO–LUMO energy gap (E_g_), Fermi level energy (E_F_) and work function (ϕ). All data are in eV.

Structure	E_H_	E_L_	E_g_	E_F_	Φ
Tyramine	− 5.80	− 0.34	5.46	− 3.07	3.07
AgNPs	− 5.23	− 2.54	2.69	− 3.88	3.88
AgNPs@tyramine-1	− 4.59	− 2.06	2.53	− 3.32	3.32
AgNPs@tyramine-2	− 4.79	− 2.17	2.62	− 3.48	3.48
AgNPs@tyramine-3	− 5.21	− 2.50	2.71	− 3.85	3.85
AgNPs@tyramine-4	− 5.07	− 2.23	2.84	− 3.65	3.65
AgNPs@tyramine-5	− 5.36	− 2.60	2.76	− 3.98	3.98

This figure infers that the HOMO is mainly located over the surface of Ag_6_ cluster and nitrogen atom (N) of tyramine rather than whole tyramine molecule. However, the LUMO is located only on the Ag_6_ cluster. Table 2 shows that the value of HOMO energy (E_H_)_,_ LUMO energy (E_L_), and energy gap (E_g_) in the most favorable configuration is computed to be − 4.59, − 2.06 and 2.53 eV respectively. The corresponding values are − 5.23 and − 2.54 eV with an energy gap of 2.69 eV for isolated Ag_6_ cluster. The magnitude of energy gap in the most favorable adsorption configuration decrease by 0.16 eV (i.e. 6%.) after tyramine adsorption. Although, this decrement in the energy gap is not remarkable but it is still noteworthy. The decrement in the magnitude of energy gap will enhance the electrical conductivity in exponential manner as energy gap is directly proportional to the electrical conductivity by the following equation^42^:

3 $${\upsigma } \propto {\mathbf{exp}} \, \left( {\frac{{ - {\varvec{E}}_{{\varvec{g}}} }}{{{\varvec{2KT}}}}} \right)$$

**Figure 3:**
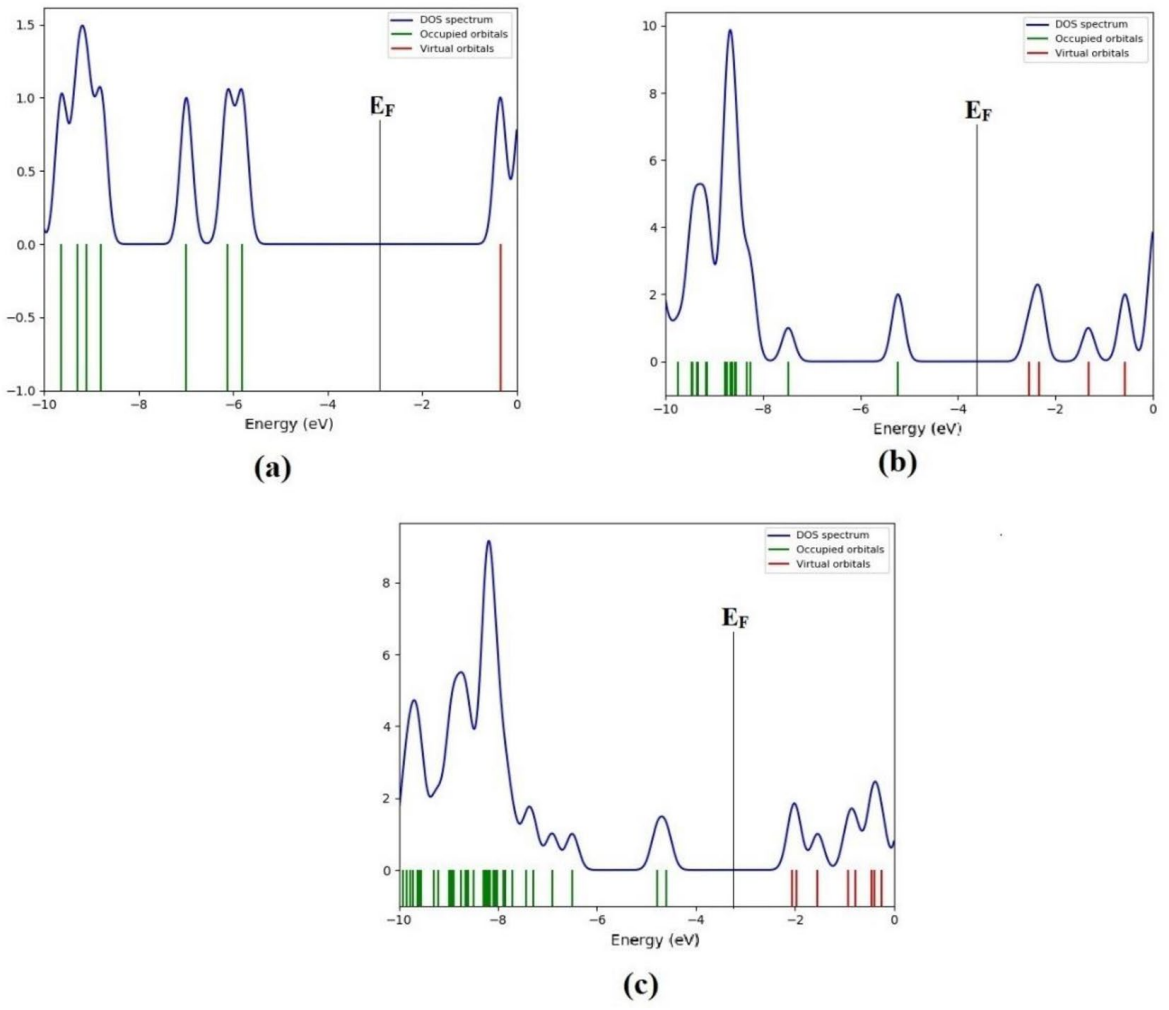
The density of states (DOS) for (**a**) Tyramine, (**b**) AgNPs and (**c**) AgNPs@tyramine.

This electrical conductivity can be converted into electrical signals to expose tyramine. The energy gap in rest configurations is tabulated in Table 2. Nhat et al.^42^ performed the interaction of thioguanine with gold cluster (Au_6_) and reported the change in energy gap by 5.9%. Moreover, the reduction in the energy gap in the present study in case of the most favorable Ag_6_@tyramine is mainly due to the interaction on nitrogen (N) atom of amine group present in the side chain of tyramine with silver atoms of Ag_6_ cluster. Total density of states (DOS) is studied in inspecting the electronic structure of tyramine on the surface silver cluster using GaussSum program^43^. Figure 3 illustrates a basic representation of the parameters of the molecular orbitals in a certain energy range and exhibits population analysis per orbital. The effect of tyramine interaction with Ag_6_ cluster on Fermi level and work function. The Fermi level is explored accordingly:

4 $$\user2{ E}_{{\varvec{F}}} = \user2{ E}_{{{\varvec{HOMO}}}} + \frac{{({\varvec{E}}_{{{\varvec{LUMO}}}} - {\varvec{E}}_{{{\varvec{HOMO}}}} )}}{2}\user2{ }$$

The calculations infer that energy of Fermi level in Ag_6_ cluster is − 3.88 eV which gets shift towards higher value in all the obtained Ag_6_@tyramine configurations except configuration-E when tyramine interacts Ag_6_ cluster. Besides, the work function (Φ = − ${E}_{F}$) of Ag_6_ cluster before tyramine adsorption is computed to be 3.88 eV. However, the adsorption of tyramine lowers the magnitude of work function which shows that the field emission properties of Ag_6_ cluster may alter upon adsorption of tyramine. Moreover, the zero point vibration energy in case of the most favorable configuration is computed to be 115.28 kcal/mol.

### Solvent effect

The effect of aqueous media on adsorption energy and electronic properties of the most favorable Ag_6_@tyraminebio-molecular conjugate is also explored using polarizable continuum model (PCM)^44^ in order to understand the impact of the real biological situation on adsorbate-adsorbent configuration. Water has been considered as solvent as it acts key role in human body. In case of aqueous phase, the adsorption energy of Ag_6_@tyramine conjugate in its most favorable configuration has been computed to be − 11.06 kcal/mol which shows strong effect on the adsorption energy of Ag_6_@tyramine conjugate in aqueous medium. Furthermore, the negative sign of adsorption energy indicates that the adsorption of tyramine over the surface of Ag_6_ cluster is of spontaneous nature. The values of dipole moment, the energy of HOMO–LUMO orbitals, energy gap and Fermi energy in the aqueous phase are tabulated in Table 3. This table reveals an increment in the energy gap between HOMO and LUMO by 0.18 eV in the presence of aqueous medium which shows that reactivity increase slightly in aqueous phase compared to the gas phase. The increased energy gap is attributed to the fact that interaction of water molecules with tyramine stabilizes HOMO and destabilizes LUMO. Dipole moment which is crucial parameter to explore the symmetricity of the molecular system and privileged magnitude of dipole moment indicates a strong interaction between the adsorbate and adsorbent. The magnitude of dipole moment in gas phase for the most favorable configuration is computed to be 5.51 Debye while it is 6.81 Debye in aqueous phase. This increment in dipole moment infers that the solubility of Ag_6_@tyramine conjugate gets enhanced by varying the medium from gas to aqueous. Additionally, the Gibbs (free) energy has been calculated using the formula below^30^:

5 $${\mathbf{DG}}^{{\mathbf{0}}} \left( {{\mathbf{298}}} \right) \, = \sum ({\varvec{\varepsilon}}_{0} + {\varvec{G}}_{{{\varvec{corr}}}} )_{{{\mathbf{final}}}} - \sum ({\varvec{\varepsilon}}_{0} + {\varvec{G}}_{{{\varvec{corr}}}} )_{{{\mathbf{Inial}}}}$$

Here, ${(\varepsilon }_{0}+{G}_{corr})$ is the total sum of electronic and thermal free energies. In gas phase Gibbs (free) energy is computed to be − 3.98 and − 0.87 kcal/mol in aqueous phase corresponding to the most favorable configuration. The negative sign indicates that adsorption of tyramine over Ag_6_ cluster is of exothermic nature.

**Table 3:** Calculated HOMO energy (E_H_), LUMO energy (E_L_), HOMO–LUMO energy gap (E_g_), Fermi level energy (E_F_) and work function (ϕ) in aqueous phase.

Structure	E_H_	E_L_	E_g_	E_F_	Φ
Tyramine	− 6.07	− 0.49	5.58	− 3.28	3.28
AgNPs	− 4.45	− 1.68	2.77	− 3.06	3.06
AgNPs@tyramine-1	− 4.29	− 1.57	2.72	− 2.93	2.93

All data are in eV.

## Recovery time

The recovery time could provide a theoretical estimation for light controlled desorption mechanism. Based on the conventional transition state theory, the recovery time can be computed using the Arrhenius-type equation^40^:

6 $$\tau = \nu_{0}^{ - 1} \exp \left( {\frac{{{-}E_{bin} }}{KT}} \right)$$

The K, υ denotes the Boltzmann’s constant and attempt frequency, respectively. The *E*_*bin*_ could be assumed as the potential barrier for the desorption process. Herein, T has been inserted at room temperature (298.15 K). The recovery times for desorption of tyramine from the studied Ag6 cluster in the presence of different light (UV, Vis, IR) are tabulated in Table 4 for the most stable configuration with adsorption energy of − 14.36 kcal/mol. The obtained recovery times in microsecond in the exposure of different light, which can provide light release control for tyramine desorption. Moreover the biosensor can be recovered rapidly.

**Table 4:** Recovery time for tyramine from Ag_6_ cluster.

Attempt frequency	UV (200–400 nm)	Vis (450–750 nm)	IR (780–1700 nm)
Conf-A	23–45 microsec	51–85 microsec	88 s–191

## NBO analysis

The second order Fock matrix was performed to determine the donor–acceptor interactions in the NBO for which we used the formula of second order perturbation approach to deduce the hyper conjugative energy (*E *^*(2)*^)^31–34^. In present study for the tyramine interaction with Ag_6_ particles, only the interaction energies of core and lone pair orbitals to anti-bonding orbitals are considered and collected in Table S1. The intramolecular interactions are formed through overlapping of bonding and anti bonding orbitals, which impel the intramolecular charge transfer (ICT) causing stabilization of the system. The required energy for the stabilization of the assorted allowed transitions within bonding and anti-bonding levels is called hyper conjugative energy. The most probable transitions have higher magnitude of hyper conjugative energy (*E*^*(2*)^) as compared to other transitions. The transitions LP1(O20)→σ*(C4-C5), LP1(O20)→π*(C4-H5), LP1(N17)→σ*(C14-H15), LP1(N17)→σ*(C14-H16) having interaction energies 4.60 kcal/mol, 25.17 kcal/mol, 5.32 kcal/mol, 1.63 kcal/mol, respectively, indicating the transition LP1(O20) →π*(C4-H5) is highly intense causing charge transfer among them. There is another intensive transition CR1(C4) → σ*(C4-O20) having energy 1.43 kcal/mol. The *E*^*(2)*^ energy for the interaction between tyramine and Ag atoms have also been collected in Table S1. There are some transitions which are very intense like LP1(N17) → LP*6(Ag25), LP1(N17) → LP*8(Ag25), σ (C14-N17) → LP*6(Ag25) with transition energy 18.66 kcal/mol, 4.92 kcal/mol and 0.89 kcal/mol, respectively. There are some other intensive interactions with Ag22, Ag23 and Ag24 with tyramine.

The occupancies of different bonding, anti-bonding, core and lone pair orbitals of carbon, nitrogen and oxygen atoms are collected in supplementary materials (see Table S2). Results indicate maximum occupancy in bonding, core and lone pair orbitals of carbon, nitrogen and oxygen atoms whereas it is very less in anti-bonding orbitals. In present investigation, the occupancy of bonding orbital of π type i.e. π(C1–C2) shows that the charge has been delocalized from this bonding orbital to its anti-bonding orbital. Moreover, the lone pair orbitals of nitrogen and oxygen LP(1) (N17) and LP(1) (O23) are calculated with 1.89e and ~ 1.98e. The occupancies of all the Ag atoms are very high close to 1e which indicates that the electrons are highly localized in Ag atoms; but when it interacts with tyramine molecule except for LP5(Ag22) which occupancy is 1.97e, i.e., this electron is resonating more.

## Conclusion

In conclusion, this research utilized the density functional theory (DFT) method to investigate the binding of tyramine to clusters of silver nanoparticle. Results could be summarized as follows:

- Tyramine binds strongly to clusters of silver nanoparticles, with adsorption energies of − 14.36 kcal/mol in the gas phase and − 11.06 kcal/mol in the aqueous phase.
- The amine group of the tyramine side chain interacts more effectively with the silver cluster compared to other sites.
- The most favorable adsorption configuration results in a 6% decrease in the energy gap, leading to increased electrical conductivity.
- The Gibbs (free) energy is negative in both the gas and aqueous phases, indicating an exothermic adsorption process.
- NBO calculations reveal a net charge transfer of 0.46e from tyramine to the silver cluster.

Consequently, these findings provide new insights into the interactions between tyramine and silver nanoparticles, laying the ground work for further research. The study's results have implications for the development of drug delivery systems, biosensors, and other formulations with enhanced efficiency. The investigation encourages future experimental studies on the functionalization of silver nanoparticle clusters with bio-molecular species.

### Supplementary Information

Supplementary Tables.

## Data Availability

All data generated or analyzed during this study are included in this published article and its supplementary information files.
