# Supplementary material for: In silico investigation on interaction of small Ag6 nano-particle cluster with tyramine neurotransmitter
Source: Sci Rep. 2023 Nov 18;13:20200. doi: 10.1038/s41598-023-45847-0 (PMC10657472; doi:10.1038/s41598-023-45847-0)
Supplement: Supplementary file 1 — Supplementary Tables. [file 41598_2023_45847_MOESM1_ESM.docx]

Table S1. Second order perturbation theory analysis.

| Donar | Acceptor | AgNPs@tyramine-1 | | |
| --- | --- | --- | --- | --- |
|  |  | E(2) | E(j)-E(i) | F(i,j) |
| Within unit 1 |  |  |  |  |
| CR1(C1) | σ*(C2 – C3) | 0.73 | 10.63 | 0.097 |
| CR1(C1) | σ*(C5 – C6) | 0.75 | 10.61 | 0.080 |
| CR1(C2) | σ*(C1 – C6) | 0.75 | 10.61 | 0.088 |
| CR1(C2) | σ*(C1 – C11) | 0.87 | 10.46 | 0.085 |
| CR1(C2) | σ*(C3 – C4) | 0.70 | 10.60 | 0.077 |
| CR1(C3) | σ*(C1 – C2) | 0.92 | 10.61 | 0.089 |
| CR1(C3) | σ*(C4 – C5) | 0.84 | 10.60 | 0.085 |
| CR1(C4) | σ*(C3- C4) | 0.60 | 10.68 | 0.075 |
| CR1(C4) | σ*(C2- C3) | 0.66 | 10.70 | 0.072 |
| CR1(C4) | σ*(C4- C5) | 0.56 | 10.68 | 0.069 |
| CR1(C4) | σ*(C5- C6) | 0.65 | 10.69 | 0.075 |
| CR1(C4) | σ*(C4- O20) | 1.43 | 10.43 | 0.110 |
| CR1(C5) | σ*(C3- C4) | 0.76 | 10.61 | 0.081 |
| CR1(C5) | σ*(C1- C6) | 0.90 | 10.62 | 0.088 |
| CR1(C5) | σ*(C4-O20) | 0.55 | 10.36 | 0.068 |
| CR1(C6) | σ*(C1 – C2) | 0.76 | 10.61 | 0.081 |
| CR1(C6) | σ*(C1 – C11) | 0.89 | 10.46 | 0.086 |
| CR1(C6) | σ*( C4 – C5) | 0.74 | 10.60 | 0.079 |
| LP1(O20) | σ*(C4- C5) | 6.60 | 1.10 | 0.076 |
| LP1(O20) | π*(C4- C5) | 25.17 | 0.34 | 0.089 |
| LP1(N17) | σ*(C14- H15) | 5.32 | 0.80 | 0.060 |
| LP1(N17) | σ*(C14- H16) | 1.63 | 0.82 | 0.033 |
| From unit 1 to unit 2 |  |  |  |  |
| σ(C6- H10) | LP*7(Ag22) | 0.13 | 0.61 | 0.009 |
| σ(C11- H13) | LP*7(Ag22) | 0.18 | 0.59 | 0.010 |
| σ(C11- C14) | LP*7(Ag22) | 0.05 | 0.70 | 0.006 |
| σ(N17- H19) | LP*9(Ag22) | 0.06 | 0.66 | 0.006 |
| LP1(N17) | LP*9(Ag22) | 0.07 | 0.35 | 0.005 |
| From unit 1 to unit 3 |  |  |  |  |
| σ(C14- H16) | LP*8(Ag24) | 0.22 | 0.63 | 0.011 |
| σ(C14- N17) | LP*7(Ag24) | 0.20 | 0.83 | 0.011 |
| σ(C14- N17) | LP*8(Ag24) | 0.23 | 0.83 | 0.012 |
| σ(N17- H19) | LP*8(Ag24) | 0.11 | 0.77 | 0.008 |
| LP1(N17) | LP*7(Ag24) | 0.23 | 0.46 | 0.009 |
| From unit 1 to unit 4 |  |  |  |  |
| σ(C1- C11) | LP*8(Ag25) | 0.13 | 0.82 | 0.009 |
| σ(C11- C14) | LP*8(Ag25) | 0.21 | 0.80 | 0.012 |
| σ(C14- N17) | LP*6(Ag25) | 0.89 | 0.69 | 0.027 |
| σ(C14- N17) | LP*8(Ag25) | 0.70 | 0.92 | 0.023 |
| LP1(N17) | LP*6(Ag25) | 18.66 | 0.32 | 0.081 |
| LP1(N17) | LP*8(Ag25) | 4.92 | 0.54 | 0.047 |

Table S2. Natural Bond Orbitals analysis.

| NBO | AgNPs@tyramine-1 | |
| --- | --- | --- |
| Orbitals | Occupancy | Energy |
| molecular unit 1 |  |  |
| σ(C1 - C2) | 1.97282 | -0.69272 |
| σ(C1 – C6) | 1.97352 | -0.69697 |
| π(C1 – C6) | 1.67375 | -0.25962 |
| σ(C1 – C11) | 1.97315 | -0.62608 |
| σ(C2 – C3) | 1.97571 | -0.69849 |
| π(C2 – C3) | 1.69470 | -0.26106 |
| σ(C3 – C4) | 1.97333 | -0.70523 |
| σ(C4 – C5) | 1.98056 | -0.71770 |
| π(C4 – C5) | 1.66528 | -0.27203 |
| σ(C5 – C6) | 1.97369 | -0.69698 |
| σ(C4 – O20) | 1.99116 | -0.88963 |
| σ(C11 – C14) | 1.97262 | -0.60724 |
| σ(C14 – N17) | 1.99279 | -0.72547 |
| σ(N17 – H18) | 1.98905 | -0.66125 |
| σ(N17 –H19) | 1.98776 | -0.66240 |
| σ(O20– H21) | 1.98931 | -0.75083 |
| σ*(C1 - C2) | 0.02694 | 0.52669 |
| σ*(C1 – C6) | 0.02591 | 0.53364 |
| π*(C1 – C6) | 0.35927 | 0.02305 |
| σ*(C1 – C11) | 0.02480 | 0.38047 |
| σ*(C2 – C3) | 0.01189 | 0.54080 |
| π*(C2 – C3) | 0.32059 | 0.02257 |
| σ*(C3 – C4) | 0.02469 | 0.52222 |
| σ*(C4 – C5) | 0.02803 | 0.52047 |
| π*(C4 – C5) | 0.38582 | 0.01192 |
| σ*(C5 – C6) | 0.01393 | 0.52848 |
| σ*(C4 – O20) | 0.02839 | 0.27021 |
| σ*(C11 – C14) | 0.02027 | 0.32816 |
| σ*(C14 – N17) | 0.01599 | 0.26489 |
| σ*(N17 – H18) | 0.01139 | 0.44484 |
| σ*(N17 –H19) | 0.00714 | 0.44458 |
| σ*(O20– H21) | 0.00878 | 0.38077 |
| CR1(C1) | 1.99905 | -10.08619 |
| CR1(C2) | 1.99907 | -10.08129 |
| CR1(C3) | 1.99909 | -10.07976 |
| CR1(C4) | 1.99883 | -10.16098 |
| CR1(C5) | 1.99910 | -10.08528 |
| CR1(C6) | 1.99909 | -10.08071 |
| CR1(C11) | 1.99924 | -10.08147 |
| CR1(C14) | 1.99925 | -10.12737 |
| CR1(O20) | 1.99985 | -18.99969 |
| CR1(N17) | 1.99968 | -14.22628 |
| LP1(N17) | 1.88762 | -0.35322 |
| LP1(O20) | 1.98146 | -0.58245 |
| LP2(O20) | 1.88826 | -0.32618 |
| Molecular unit 2 |  |  |
| CR1(Ag22) | 1.99144 | -3.53420 |
| CR2(Ag22) | 1.99948 | -2.23698 |
| CR3(Ag22) | 1.99971 | -2.23768 |
| CR4(Ag22) | 1.99877 | -2.23687 |
| LP1(Ag22) | 1.99685 | -0.32818 |
| LP2(Ag22) | 1.99676 | -0.32642 |
| LP3(Ag22) | 1.99608 | -0.32702 |
| LP4(Ag22) | 1.98040 | -0.32918 |
| LP5(Ag22) | 1.97788 | -0.32967 |
|  |  |  |
| CR1(Ag24) | 1.99388 | -3.52200 |
| CR2(Ag24) | 1.99932 | -2.22077 |
| CR3(Ag24) | 1.99871 | -2.22179 |
| CR4(Ag24) | 1.99925 | -2.22070 |
| LP1(Ag24) | 1.99857 | -0.30984 |
| LP2(Ag24) | 1.99670 | -0.30946 |
| LP3(Ag24) | 1.99578 | -0.31149 |
| LP4(Ag24) | 1.99488 | -0.31277 |
| LP5(Ag24) | 1.98831 | -0.31339 |
|  |  |  |
| CR1(Ag25) | 1.99281 | -3.53282 |
| CR2(Ag25) | 1.99927 | -2.22298 |
| CR3(Ag25) | 1.99957 | -2.22577 |
| CR4(Ag25) | 1.99921 | -2.22544 |
| LP1(Ag25) | 1.99748 | -0.31431 |
| LP2(Ag25) | 1.99701 | -0.31490 |
| LP3(Ag25) | 1.99454 | -0.31462 |
| LP4(Ag25) | 1.99376 | -0.31553 |
| LP5(Ag25) | 1.98374 | -0.31333 |
